# 2,9-Dimethyl-7-phenyl-*N*-(4-methyl­phen­yl)dibenzo[*b*,*h*][1,6]naphthyridin-6-amine

**DOI:** 10.1107/S1600536810051196

**Published:** 2010-12-11

**Authors:** K. N. Vennila, M. Manoj, K. Prabha, K. J. Rajendra Prasad, D. Velmurugan

**Affiliations:** a Centre of Advanced Study in Crystallography and Biophysics, University of Madras, Guindy Campus, Chennai 600 025, India; b Department of Chemistry, Bharathiar University, Coimbatore 641 046, India

## Abstract

The title compound, C_31_H_25_N_3_, was synthesized from 6,4′,4′′-trimethyl-2,4-bis­(*N*-phenyl­amino)­quinoline and is the first structural example containing a phenyl and phenyl­amino fragment attached to a fused dibenzo[1,6]naphthyridine moiety. The fused tetra­cyclic ring system is essentially planar [r.m.s. deviation = 0.08 (3) Å]. The phenyl ring and the phenyl­amino group are inclined by 82.68 (6) and 35.31 (5)°, respectively, to the mean plane of the fused tetra­cyclic ring system. A weak intra­molecular N—H⋯π(arene) inter­action may in part influence the conformation of the mol­ecule. In the crystal, mol­ecules are linked by weak inter­molecular C—H⋯N hydrogen bonds into centrosymmetric dimers. Additional stabilization is provided by weak C—H⋯π and π–π stacking inter­actions [centroid–centroid distances = 3.834 (2) and 3.898 (1) Å].

## Related literature

For the biological activity of [1,6]naphthyridine derivatives, see: Ruchelman *et al.* (2003 ▶, 2005 ▶); Hinschberger *et al.* (2003 ▶); Bedard *et al.* (2000 ▶); Feng *et al.* (2008 ▶). For the synthesis of the title compound, see: Manoj & Rajendra Prasad (2009 ▶). For the crystal structures of other [1,6]naphthrydine derivatives, see: Peng *et al.* (2009 ▶); Sivakumar *et al.* (2003 ▶); Seebacher *et al.* (2010 ▶); Vennila *et al.* (2010 ▶). For bond-length data, see: Allen *et al.* (1987 ▶).
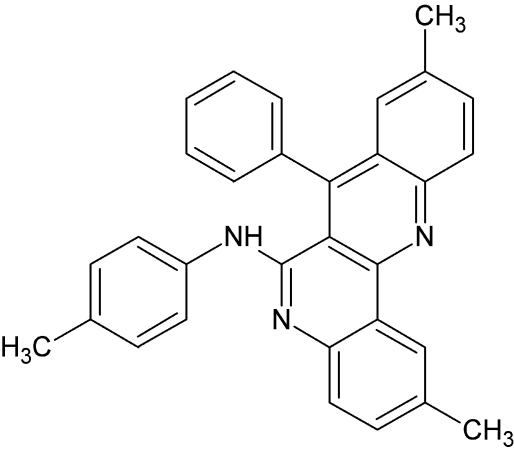


## Experimental

### 

#### Crystal data

C_31_H_25_N_3_
*M* *_r_* = 439.54
Monoclinic, 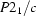

*a* = 11.9390 (4) Å
*b* = 10.5595 (4) Å
*c* = 19.6084 (7) Å
β = 107.369 (2)°
*V* = 2359.31 (15) Å^3^
*Z* = 4
Mo *K*α radiation
μ = 0.07 mm^−1^
*T* = 293 K
0.30 × 0.20 × 0.20 mm

#### Data collection

Bruker APEXII CCD diffractometer
Absorption correction: multi-scan (*SADABS*; Bruker, 2004 ▶) *T*_min_ = 0.982, *T*_max_ = 0.986
17909 measured reflections
2938 independent reflections
2197 reflections with *I* > 2σ(*I*)
*R*_int_ = 0.037
θ_max_ = 22.1°

#### Refinement

*R*[*F* ^2^ > 2σ(*F* ^2^)] = 0.042
*wR*(*F* ^2^) = 0.132
*S* = 1.07
2938 reflections
310 parameters
H-atom parameters constrained
Δρ_max_ = 0.21 e Å^−3^
Δρ_min_ = −0.23 e Å^−3^

### 

Data collection: *APEX2* (Bruker, 2004 ▶); cell refinement: *APEX2* and *SAINT* (Bruker, 2004 ▶); data reduction: *SAINT*; program(s) used to solve structure: *SHELXS97* (Sheldrick, 2008 ▶); program(s) used to refine structure: *SHELXL97* (Sheldrick, 2008 ▶); molecular graphics: *ORTEPII* (Johnson, 1976) ▶; software used to prepare material for publication: *SHELXL97* and *PLATON* (Spek, 2009 ▶).

## Supplementary Material

Crystal structure: contains datablocks global, I. DOI: 10.1107/S1600536810051196/lh5177sup1.cif

Structure factors: contains datablocks I. DOI: 10.1107/S1600536810051196/lh5177Isup2.hkl

Additional supplementary materials:  crystallographic information; 3D view; checkCIF report

## Figures and Tables

**Table 1:** Hydrogen-bond geometry (Å, °) *Cg*1 and *Cg*2 are the centroids of the C17–C22 and C23–C28 rings, respectively.

*D*—H⋯*A*	*D*—H	H⋯*A*	*D*⋯*A*	*D*—H⋯*A*
N3—H3⋯*Cg*1	0.86	2.51	3.364 (3)	174
C18—H18⋯*Cg*2^i^	0.93	2.62	3.495 (3)	156
C29—H29*B*⋯*Cg*2^ii^	0.96	2.90	3.622 (3)	133
C22—H22⋯N2^iii^	0.93	2.51	3.419 (3)	166

Symmetry codes: (i) 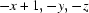; (ii) 
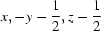
; (iii) 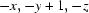.

**Fig. 1.**
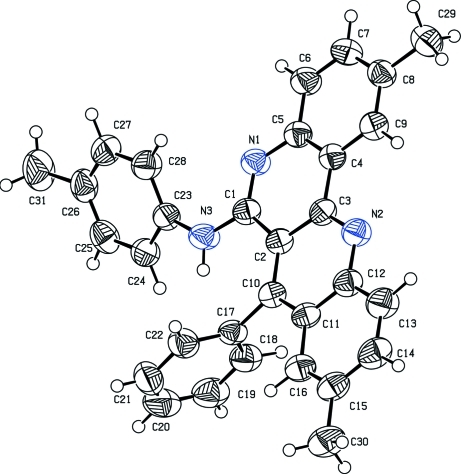
The molecular structure of the title compound showing the thermal ellipsoids drawn at the 50% probability level.

**Fig. 2.**
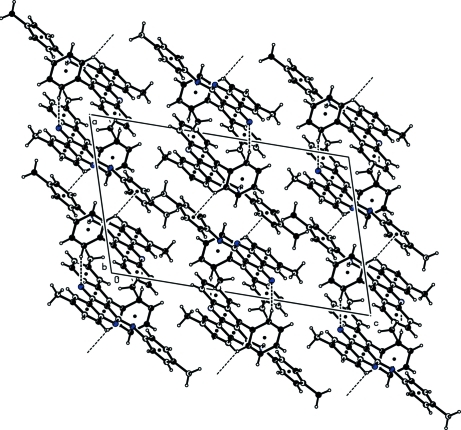
The crystal packing viewed along the b-axis with weak hydrogen bonds and C-H..π interactions shown as dotted lines

## supplementary crystallographic information

### Comment

The crystal structures of number of differently substituted naphthyridine derivatives have already been reported  (e.g. Peng  *et al.* 2009) but among those only few are dibenzo[1,6] naphthyridine compounds (e.g. Sivakumar *et al.* 2003; Seebacher *et al.*, 2010). [1,6]Naphthyridine compounds are known to have biological activities (Ruchelman *et al.* 2005; Hinschberger *et al.* 2003; Feng *et al.*, 2008). Dibenzo[1,6]naphthyridines are reported to have potent Topomerase I targeting, cytotoxic (Ruchelman *et al.*, 2003) and are also proven anti-tumour agents. A series of [1,6] naphthyridine compounds were shown to exhibit potent activity against human cytomegalovirus (Bedard *et al.* , 2000). We are focused on preparing heterocyclic naphthyridine derivatives with potential biological properties. The crystal structure of the title compound is presented herein.

The molecular structure of the title compound is shown in Fig. 1 The fused tetracyclic ring system is essentially planar (r.m.s. deviation = 0.08 (3)Å) as was reported for a previously determined structure (Vennila *et al.*, 2010). The phenyl ring and the phenyl amino group are inclined by 82.68 (6)° and 35.31 (5)°, respectively to the mean plane of the fused tetracyclic ring system. The bond lengths (Allen *et al.*, 1987) and angles are in the normal ranges. In the crystal structure (Fig. 2), molecules are linked by weak intermolecular C-H···N hydrogen bonds into centrosymmetric dimers. Additional stabilization is provided by weak C-H···π and π–π stacking interactions.

### Experimental

The synthesis follows the procedure of Manoj & Rajendra Prasad (2009). *Preparation of 6,4',4''-Trimethyl-2,4-bis-(N-phenylamino)quinoline*: A mixture of appropriate 2,4-dichloro-6-methylquinoline (0.010 mol) and *p*-toluidine (0.010 mol) was heated at 433K for half an hour. The product obtained was washed with water, dried and purified by column chromatography over silica gel and eluted with ethyl acetate : methanol mixture (95 : 5) to yield a white solid. The product was recrytallized from methanol. *Preparation of 2,9,4'-Trimethyl-7-phenyl-6-(N-phenylamino) dibenzo[*b,h*][1,6] naphthyridine*: An mixture of 6,4',4''-trimethyl-2,4-bis-(N-phenylamino) quinoline (0.0010 mol) and benzoic acid (0.0011 mol) was added to polyphosphoric acid (3 g of P_2_O_5_ in 1.5 mL of H_3_PO_4_) and kept at 323-328 K for 5 hours. The reaction was monitored by TLC. After the completion of the reaction, the reaction mixture was poured into ice water and neutralised with saturated NaHCO_3_ solution to remove the excess benzoic acid. The precipitate was filtered, dried and purified by column chromatography over silica gel using petroleum ether : ethyl acetate (99 : 1) mixture to obtain yellow solid. The yellow solid was dissolved in ethylacetate and left to crystallize at 277K for about 6 months. Only few crystals were obtained and the best available was used for the X-ray structure analysis.

### Refinement

The H-atoms were positioned geometrically and treated as riding atoms: C—H =0.93 Å H-aromatic, C—H = 0.96 Å H-methyl, and N—H = 0.86 Å, with *U*_iso_ = k×*U*_eq_(parent C or N-atom), where k = 1.5 for methyl H-atoms, and = 1.2 for all other H-atoms. The low percentage of data used (because of low theta cut-off) may affect the precision of the structure.

### Crystal data

**Table:**

C_31_H_25_N_3_	*F*(000) = 928
*M**_r_* = 439.54	*D*_x_ = 1.237 Mg m^−^^3^
Monoclinic, *P*2_1_/*c*	Mo *K*α radiation, λ = 0.71073 Å
Hall symbol: -P 2ybc	Cell parameters from 2938 reflections
*a* = 11.9390 (4) Å	θ = 2.2–21.2°
*b* = 10.5595 (4) Å	µ = 0.07 mm^−^^1^
*c* = 19.6084 (7) Å	*T* = 293 K
β = 107.369 (2)°	Prismatic, yellow
*V* = 2359.31 (15) Å^3^	0.30 × 0.20 × 0.20 mm
*Z* = 4	

### Data collection

**Table:**

Bruker APEXII CCD diffractometer	2938 independent reflections
Radiation source: fine-focus sealed tube	2197 reflections with *I* > 2σ(*I*)
graphite	*R*_int_ = 0.037
ω and φ scans	θ_max_ = 22.1°, θ_min_ = 1.8°
Absorption correction: multi-scan (*SADABS*; Bruker, 2004)	*h* = −12→12
*T*_min_ = 0.982, *T*_max_ = 0.986	*k* = −11→11
17909 measured reflections	*l* = −20→20

### Refinement

**Table:**

Refinement on *F*^2^	Primary atom site location: structure-invariant direct methods
Least-squares matrix: full	Secondary atom site location: difference Fourier map
*R*[*F*^2^ > 2σ(*F*^2^)] = 0.042	Hydrogen site location: inferred from neighbouring sites
*wR*(*F*^2^) = 0.132	H-atom parameters constrained
*S* = 1.07	*w* = 1/[σ^2^(*F*_o_^2^) + (0.0713*P*)^2^ + 0.4411*P*] where *P* = (*F*_o_^2^ + 2*F*_c_^2^)/3
2938 reflections	(Δ/σ)_max_ = 0.034
310 parameters	Δρ_max_ = 0.21 e Å^−^^3^
0 restraints	Δρ_min_ = −0.23 e Å^−^^3^

### Special details

**Table:**

Geometry. All esds (except the esd in the dihedral angle between two l.s. planes) are estimated using the full covariance matrix. The cell esds are taken into account individually in the estimation of esds in distances, angles and torsion angles; correlations between esds in cell parameters are only used when they are defined by crystal symmetry. An approximate (isotropic) treatment of cell esds is used for estimating esds involving l.s. planes.
Refinement. Refinement of F^2^ against ALL reflections. The weighted R-factor wR and goodness of fit S are based on F^2^, conventional R-factors R are based on F, with F set to zero for negative F^2^. The threshold expression of F^2^ > 2sigma(F^2^) is used only for calculating R-factors(gt) etc. and is not relevant to the choice of reflections for refinement. R-factors based on F^2^ are statistically about twice as large as those based on F, and R- factors based on ALL data will be even larger.

### Fractional atomic coordinates and isotropic or equivalent isotropic displacement parameters (Å^2^)

**Table:**

	*x*	*y*	*z*	*U*_iso_*/*U*_eq_	
C2	0.21879 (18)	0.4613 (2)	0.03581 (11)	0.0434 (6)	
N1	0.32355 (16)	0.27439 (18)	0.01274 (10)	0.0508 (5)	
N2	0.12176 (16)	0.44039 (18)	0.12785 (10)	0.0529 (5)	
C3	0.18596 (18)	0.3921 (2)	0.08942 (12)	0.0448 (6)	
C4	0.22420 (18)	0.2617 (2)	0.10461 (12)	0.0448 (6)	
C12	0.08099 (19)	0.5597 (2)	0.11381 (12)	0.0511 (6)	
N3	0.32400 (17)	0.45075 (18)	−0.05365 (10)	0.0569 (6)	
H3	0.3031	0.5288	−0.0607	0.068*	
C11	0.10670 (18)	0.6366 (2)	0.06154 (12)	0.0461 (6)	
C5	0.29150 (19)	0.2088 (2)	0.06518 (12)	0.0473 (6)	
C1	0.29049 (18)	0.3908 (2)	−0.00132 (12)	0.0459 (6)	
C10	0.18035 (18)	0.5874 (2)	0.02346 (11)	0.0448 (6)	
C9	0.19467 (19)	0.1887 (2)	0.15606 (12)	0.0512 (6)	
H9	0.1499	0.2250	0.1824	0.061*	
C8	0.2296 (2)	0.0654 (2)	0.16883 (12)	0.0523 (6)	
C17	0.21691 (19)	0.6767 (2)	−0.02498 (13)	0.0464 (6)	
C23	0.38787 (19)	0.4030 (2)	−0.09768 (12)	0.0480 (6)	
C16	0.0570 (2)	0.7604 (2)	0.05065 (13)	0.0546 (6)	
H16	0.0741	0.8128	0.0169	0.066*	
C6	0.3278 (2)	0.0832 (2)	0.07839 (13)	0.0592 (7)	
H6	0.3736	0.0465	0.0528	0.071*	
C14	−0.0376 (2)	0.7257 (3)	0.13967 (14)	0.0659 (7)	
H14	−0.0858	0.7554	0.1657	0.079*	
C13	0.0083 (2)	0.6078 (3)	0.15287 (14)	0.0666 (7)	
H13	−0.0082	0.5581	0.1879	0.080*	
C7	0.2968 (2)	0.0136 (2)	0.12858 (13)	0.0604 (7)	
H7	0.3210	−0.0702	0.1361	0.073*	
C24	0.4578 (2)	0.4875 (2)	−0.11974 (13)	0.0548 (6)	
H24	0.4660	0.5699	−0.1022	0.066*	
C15	−0.0144 (2)	0.8045 (2)	0.08782 (14)	0.0575 (7)	
C29	0.1979 (2)	−0.0121 (3)	0.22479 (14)	0.0678 (8)	
H29A	0.1430	0.0340	0.2424	0.102*	
H29B	0.2674	−0.0292	0.2635	0.102*	
H29C	0.1632	−0.0906	0.2042	0.102*	
C22	0.1465 (2)	0.7020 (2)	−0.09377 (14)	0.0617 (7)	
H22	0.0736	0.6633	−0.1112	0.074*	
C28	0.3782 (2)	0.2810 (2)	−0.12414 (13)	0.0571 (7)	
H28	0.3323	0.2220	−0.1094	0.069*	
C27	0.4362 (2)	0.2465 (3)	−0.17227 (13)	0.0632 (7)	
H27	0.4281	0.1641	−0.1898	0.076*	
C26	0.5057 (2)	0.3298 (3)	−0.19527 (13)	0.0617 (7)	
C25	0.5157 (2)	0.4509 (3)	−0.16770 (13)	0.0609 (7)	
H25	0.5628	0.5093	−0.1818	0.073*	
C18	0.3242 (2)	0.7371 (2)	−0.00024 (15)	0.0591 (7)	
H18	0.3714	0.7220	0.0462	0.071*	
C21	0.1851 (3)	0.7849 (3)	−0.13643 (16)	0.0780 (9)	
H21	0.1380	0.8018	−0.1827	0.094*	
C30	−0.0682 (2)	0.9343 (2)	0.07514 (16)	0.0744 (8)	
H30A	−0.0115	0.9959	0.0999	0.112*	
H30B	−0.1353	0.9375	0.0925	0.112*	
H30C	−0.0921	0.9525	0.0249	0.112*	
C20	0.2927 (3)	0.8426 (3)	−0.1109 (2)	0.0818 (10)	
H20	0.3183	0.8980	−0.1399	0.098*	
C19	0.3623 (3)	0.8190 (3)	−0.04288 (19)	0.0752 (8)	
H19	0.4351	0.8582	−0.0257	0.090*	
C31	0.5664 (3)	0.2936 (3)	−0.24940 (15)	0.0941 (11)	
H31A	0.5255	0.3305	−0.2948	0.141*	
H31B	0.5666	0.2031	−0.2539	0.141*	
H31C	0.6457	0.3242	−0.2341	0.141*	

### Atomic displacement parameters (Å^2^)

**Table:**

	*U*^11^	*U*^22^	*U*^33^	*U*^12^	*U*^13^	*U*^23^
C2	0.0396 (12)	0.0431 (14)	0.0481 (14)	0.0009 (10)	0.0139 (11)	−0.0022 (11)
N1	0.0549 (12)	0.0442 (13)	0.0578 (13)	0.0076 (9)	0.0240 (10)	0.0019 (10)
N2	0.0570 (12)	0.0496 (13)	0.0591 (13)	0.0049 (10)	0.0277 (10)	0.0003 (10)
C3	0.0415 (13)	0.0463 (14)	0.0475 (14)	0.0003 (10)	0.0149 (11)	−0.0041 (11)
C4	0.0427 (13)	0.0444 (14)	0.0461 (14)	−0.0006 (10)	0.0115 (11)	−0.0010 (11)
C12	0.0506 (14)	0.0493 (16)	0.0578 (15)	0.0032 (12)	0.0228 (12)	−0.0040 (13)
N3	0.0722 (14)	0.0440 (12)	0.0677 (14)	0.0125 (10)	0.0410 (12)	0.0060 (10)
C11	0.0436 (13)	0.0410 (14)	0.0549 (15)	0.0023 (10)	0.0165 (11)	−0.0046 (11)
C5	0.0470 (13)	0.0435 (15)	0.0508 (14)	0.0027 (11)	0.0138 (11)	−0.0007 (12)
C1	0.0434 (13)	0.0448 (15)	0.0507 (15)	0.0025 (11)	0.0158 (11)	−0.0020 (12)
C10	0.0422 (13)	0.0435 (15)	0.0485 (14)	0.0001 (10)	0.0133 (11)	−0.0023 (11)
C9	0.0489 (14)	0.0526 (17)	0.0526 (15)	0.0011 (11)	0.0158 (12)	−0.0003 (12)
C8	0.0520 (14)	0.0495 (16)	0.0509 (15)	−0.0005 (12)	0.0088 (12)	0.0042 (12)
C17	0.0485 (14)	0.0387 (14)	0.0565 (16)	0.0069 (11)	0.0225 (12)	0.0006 (12)
C23	0.0508 (14)	0.0473 (15)	0.0492 (14)	0.0090 (11)	0.0198 (12)	0.0027 (12)
C16	0.0553 (14)	0.0475 (15)	0.0620 (16)	0.0045 (11)	0.0189 (13)	−0.0021 (12)
C6	0.0664 (16)	0.0510 (17)	0.0650 (17)	0.0116 (13)	0.0266 (14)	0.0006 (13)
C14	0.0642 (16)	0.0645 (19)	0.080 (2)	0.0080 (14)	0.0376 (15)	−0.0138 (15)
C13	0.0743 (18)	0.0638 (19)	0.0756 (18)	0.0079 (14)	0.0437 (15)	−0.0024 (15)
C7	0.0693 (17)	0.0465 (16)	0.0641 (18)	0.0089 (12)	0.0178 (14)	0.0072 (13)
C24	0.0616 (15)	0.0505 (16)	0.0568 (16)	0.0037 (12)	0.0244 (13)	0.0030 (12)
C15	0.0507 (14)	0.0545 (16)	0.0683 (17)	0.0057 (12)	0.0193 (13)	−0.0115 (14)
C29	0.0722 (18)	0.0643 (18)	0.0665 (18)	−0.0023 (13)	0.0200 (14)	0.0124 (14)
C22	0.0666 (16)	0.0567 (17)	0.0641 (18)	0.0094 (13)	0.0230 (15)	0.0042 (14)
C28	0.0601 (15)	0.0553 (17)	0.0623 (16)	0.0010 (12)	0.0280 (13)	−0.0026 (13)
C27	0.0730 (17)	0.0601 (17)	0.0597 (17)	0.0098 (14)	0.0248 (14)	−0.0055 (13)
C26	0.0681 (17)	0.071 (2)	0.0508 (16)	0.0211 (14)	0.0254 (13)	0.0070 (14)
C25	0.0569 (15)	0.073 (2)	0.0594 (16)	0.0070 (13)	0.0274 (13)	0.0154 (15)
C18	0.0631 (16)	0.0503 (16)	0.0694 (17)	−0.0022 (13)	0.0282 (14)	−0.0012 (13)
C21	0.111 (3)	0.070 (2)	0.0632 (19)	0.0262 (19)	0.0413 (18)	0.0143 (16)
C30	0.0693 (17)	0.0624 (18)	0.091 (2)	0.0197 (14)	0.0233 (15)	−0.0106 (16)
C20	0.118 (3)	0.0528 (19)	0.105 (3)	0.0048 (18)	0.079 (2)	0.0082 (18)
C19	0.084 (2)	0.0567 (19)	0.101 (3)	−0.0073 (15)	0.052 (2)	−0.0060 (18)
C31	0.115 (3)	0.111 (3)	0.076 (2)	0.033 (2)	0.058 (2)	0.0119 (19)

### Geometric parameters (Å, °)

**Table:**

C2—C10	1.406 (3)	C14—C15	1.403 (3)
C2—C3	1.428 (3)	C14—H14	0.9300
C2—C1	1.480 (3)	C13—H13	0.9300
N1—C1	1.295 (3)	C7—H7	0.9300
N1—C5	1.385 (3)	C24—C25	1.379 (3)
N2—C3	1.327 (3)	C24—H24	0.9300
N2—C12	1.349 (3)	C15—C30	1.503 (3)
C3—C4	1.453 (3)	C29—H29A	0.9600
C4—C5	1.388 (3)	C29—H29B	0.9600
C4—C9	1.396 (3)	C29—H29C	0.9600
C12—C11	1.411 (3)	C22—C21	1.381 (4)
C12—C13	1.412 (3)	C22—H22	0.9300
N3—C1	1.364 (3)	C28—C27	1.376 (3)
N3—C23	1.405 (3)	C28—H28	0.9300
N3—H3	0.8600	C27—C26	1.374 (4)
C11—C10	1.412 (3)	C27—H27	0.9300
C11—C16	1.424 (3)	C26—C25	1.380 (4)
C5—C6	1.396 (3)	C26—C31	1.502 (3)
C10—C17	1.493 (3)	C25—H25	0.9300
C9—C8	1.368 (3)	C18—C19	1.371 (4)
C9—H9	0.9300	C18—H18	0.9300
C8—C7	1.394 (3)	C21—C20	1.374 (4)
C8—C29	1.505 (3)	C21—H21	0.9300
C17—C18	1.383 (3)	C30—H30A	0.9600
C17—C22	1.386 (3)	C30—H30B	0.9600
C23—C24	1.377 (3)	C30—H30C	0.9600
C23—C28	1.381 (3)	C20—C19	1.367 (4)
C16—C15	1.359 (3)	C20—H20	0.9300
C16—H16	0.9300	C19—H19	0.9300
C6—C7	1.365 (3)	C31—H31A	0.9600
C6—H6	0.9300	C31—H31B	0.9600
C14—C13	1.354 (3)	C31—H31C	0.9600
			
C10—C2—C3	117.67 (19)	C6—C7—H7	119.2
C10—C2—C1	126.8 (2)	C8—C7—H7	119.2
C3—C2—C1	115.5 (2)	C23—C24—C25	120.4 (2)
C1—N1—C5	119.88 (18)	C23—C24—H24	119.8
C3—N2—C12	118.52 (19)	C25—C24—H24	119.8
N2—C3—C2	123.6 (2)	C16—C15—C14	118.5 (2)
N2—C3—C4	116.57 (19)	C16—C15—C30	121.9 (2)
C2—C3—C4	119.82 (18)	C14—C15—C30	119.6 (2)
C5—C4—C9	119.6 (2)	C8—C29—H29A	109.5
C5—C4—C3	117.8 (2)	C8—C29—H29B	109.5
C9—C4—C3	122.7 (2)	H29A—C29—H29B	109.5
N2—C12—C11	122.84 (19)	C8—C29—H29C	109.5
N2—C12—C13	117.9 (2)	H29A—C29—H29C	109.5
C11—C12—C13	119.3 (2)	H29B—C29—H29C	109.5
C1—N3—C23	129.1 (2)	C21—C22—C17	119.8 (3)
C1—N3—H3	115.4	C21—C22—H22	120.1
C23—N3—H3	115.4	C17—C22—H22	120.1
C10—C11—C12	118.5 (2)	C27—C28—C23	120.1 (2)
C10—C11—C16	123.7 (2)	C27—C28—H28	119.9
C12—C11—C16	117.8 (2)	C23—C28—H28	119.9
N1—C5—C4	123.1 (2)	C28—C27—C26	122.2 (3)
N1—C5—C6	118.3 (2)	C28—C27—H27	118.9
C4—C5—C6	118.6 (2)	C26—C27—H27	118.9
N1—C1—N3	117.50 (19)	C27—C26—C25	117.1 (2)
N1—C1—C2	123.9 (2)	C27—C26—C31	122.4 (3)
N3—C1—C2	118.6 (2)	C25—C26—C31	120.6 (3)
C2—C10—C11	118.7 (2)	C24—C25—C26	121.7 (2)
C2—C10—C17	124.41 (19)	C24—C25—H25	119.2
C11—C10—C17	116.81 (19)	C26—C25—H25	119.2
C8—C9—C4	122.0 (2)	C19—C18—C17	121.2 (3)
C8—C9—H9	119.0	C19—C18—H18	119.4
C4—C9—H9	119.0	C17—C18—H18	119.4
C9—C8—C7	117.7 (2)	C20—C21—C22	120.3 (3)
C9—C8—C29	121.5 (2)	C20—C21—H21	119.9
C7—C8—C29	120.8 (2)	C22—C21—H21	119.9
C18—C17—C22	118.8 (2)	C15—C30—H30A	109.5
C18—C17—C10	119.0 (2)	C15—C30—H30B	109.5
C22—C17—C10	122.3 (2)	H30A—C30—H30B	109.5
C24—C23—C28	118.5 (2)	C15—C30—H30C	109.5
C24—C23—N3	116.8 (2)	H30A—C30—H30C	109.5
C28—C23—N3	124.4 (2)	H30B—C30—H30C	109.5
C15—C16—C11	122.3 (2)	C19—C20—C21	120.3 (3)
C15—C16—H16	118.9	C19—C20—H20	119.8
C11—C16—H16	118.9	C21—C20—H20	119.8
C7—C6—C5	120.6 (2)	C20—C19—C18	119.6 (3)
C7—C6—H6	119.7	C20—C19—H19	120.2
C5—C6—H6	119.7	C18—C19—H19	120.2
C13—C14—C15	121.8 (2)	C26—C31—H31A	109.5
C13—C14—H14	119.1	C26—C31—H31B	109.5
C15—C14—H14	119.1	H31A—C31—H31B	109.5
C14—C13—C12	120.4 (2)	C26—C31—H31C	109.5
C14—C13—H13	119.8	H31A—C31—H31C	109.5
C12—C13—H13	119.8	H31B—C31—H31C	109.5
C6—C7—C8	121.6 (2)		
			
C12—N2—C3—C2	2.8 (3)	C4—C9—C8—C7	0.2 (3)
C12—N2—C3—C4	−177.8 (2)	C4—C9—C8—C29	179.5 (2)
C10—C2—C3—N2	−0.1 (3)	C2—C10—C17—C18	−80.6 (3)
C1—C2—C3—N2	−179.91 (19)	C11—C10—C17—C18	96.2 (3)
C10—C2—C3—C4	−179.53 (19)	C2—C10—C17—C22	100.0 (3)
C1—C2—C3—C4	0.6 (3)	C11—C10—C17—C22	−83.2 (3)
N2—C3—C4—C5	180.0 (2)	C1—N3—C23—C24	−149.1 (2)
C2—C3—C4—C5	−0.5 (3)	C1—N3—C23—C28	36.0 (4)
N2—C3—C4—C9	0.8 (3)	C10—C11—C16—C15	179.4 (2)
C2—C3—C4—C9	−179.7 (2)	C12—C11—C16—C15	−1.0 (3)
C3—N2—C12—C11	−2.0 (3)	N1—C5—C6—C7	−178.8 (2)
C3—N2—C12—C13	177.1 (2)	C4—C5—C6—C7	0.6 (4)
N2—C12—C11—C10	−1.4 (3)	C15—C14—C13—C12	−0.5 (4)
C13—C12—C11—C10	179.5 (2)	N2—C12—C13—C14	−178.3 (2)
N2—C12—C11—C16	179.0 (2)	C11—C12—C13—C14	0.9 (4)
C13—C12—C11—C16	−0.1 (3)	C5—C6—C7—C8	−0.9 (4)
C1—N1—C5—C4	0.0 (3)	C9—C8—C7—C6	0.4 (4)
C1—N1—C5—C6	179.4 (2)	C29—C8—C7—C6	−178.9 (2)
C9—C4—C5—N1	179.4 (2)	C28—C23—C24—C25	0.6 (3)
C3—C4—C5—N1	0.2 (3)	N3—C23—C24—C25	−174.7 (2)
C9—C4—C5—C6	0.0 (3)	C11—C16—C15—C14	1.4 (4)
C3—C4—C5—C6	−179.2 (2)	C11—C16—C15—C30	−178.9 (2)
C5—N1—C1—N3	−179.7 (2)	C13—C14—C15—C16	−0.6 (4)
C5—N1—C1—C2	0.1 (3)	C13—C14—C15—C30	179.7 (2)
C23—N3—C1—N1	2.3 (4)	C18—C17—C22—C21	1.0 (3)
C23—N3—C1—C2	−177.5 (2)	C10—C17—C22—C21	−179.6 (2)
C10—C2—C1—N1	179.7 (2)	C24—C23—C28—C27	−0.9 (4)
C3—C2—C1—N1	−0.5 (3)	N3—C23—C28—C27	173.9 (2)
C10—C2—C1—N3	−0.4 (3)	C23—C28—C27—C26	0.5 (4)
C3—C2—C1—N3	179.37 (19)	C28—C27—C26—C25	0.3 (4)
C3—C2—C10—C11	−3.4 (3)	C28—C27—C26—C31	−178.2 (2)
C1—C2—C10—C11	176.4 (2)	C23—C24—C25—C26	0.2 (4)
C3—C2—C10—C17	173.4 (2)	C27—C26—C25—C24	−0.7 (4)
C1—C2—C10—C17	−6.8 (4)	C31—C26—C25—C24	177.8 (2)
C12—C11—C10—C2	4.1 (3)	C22—C17—C18—C19	−1.2 (4)
C16—C11—C10—C2	−176.3 (2)	C10—C17—C18—C19	179.3 (2)
C12—C11—C10—C17	−172.9 (2)	C17—C22—C21—C20	−0.2 (4)
C16—C11—C10—C17	6.6 (3)	C22—C21—C20—C19	−0.3 (4)
C5—C4—C9—C8	−0.4 (3)	C21—C20—C19—C18	0.1 (4)
C3—C4—C9—C8	178.8 (2)	C17—C18—C19—C20	0.7 (4)

### Hydrogen-bond geometry (Å, °)

**Table:**

Cg1 and Cg2 are the centroids of the C17–C22 and C23–C28 rings, respectively.

**Table:**

*D*—H···*A*	*D*—H	H···*A*	*D*···*A*	*D*—H···*A*
N3—H3···Cg1	0.86	2.51	3.364 (3)	174
C18—H18···Cg2^i^	0.93	2.62	3.495 (3)	156
C29—H29B···Cg2^ii^	0.96	2.90	3.622 (3)	133
C22—H22···N2^iii^	0.93	2.51	3.419 (3)	166
C28—H28···N1	0.93	2.49	2.946 (3)	110

Symmetry codes: (i) −*x*+1, −*y*, −*z*; (ii) *x*, −*y*−1/2, *z*−1/2; (iii) −*x*, −*y*+1, −*z*.

### Table 2 π–π interactions [Å]

Cg2, Cg3 and Cg4 are the centroids of the N1/C1–C5, N2/C2/C3/C10–C12 and C11–C16 rings, respectively.

**Table:**

Cg(I)	Cg(J)	Centroid-to-Centroid
Cg(2)	Cg(4^i^)	3.834 (2)
Cg(3)	Cg(3^i^)	3.898 (1)

Symmetry code (i): -x, 1-y, -z.
